# Diagnostic Performance of Biomarker-Based Scores as Predictors of Metabolic Dysfunction-Associated Fatty Liver Disease Risk in Healthy Children

**DOI:** 10.3390/nu15163667

**Published:** 2023-08-21

**Authors:** Katarzyna Bergmann, Anna Stefanska, Magdalena Krintus, Lukasz Szternel, Wojciech J. Bilinski, Przemyslaw T. Paradowski, Grazyna Sypniewska

**Affiliations:** 1 Department of Laboratory Medicine, Collegium Medicum in Bydgoszcz, Nicolaus Copernicus University, 87-100 Torun, Poland; zuzanna@cm.umk.pl (A.S.); krintus@cm.umk.pl (M.K.); l.szternel@cm.umk.pl (L.S.); odes@cm.umk.pl (G.S.); 2 Department of Orthopaedics, KoMed Poddebice Health Center, 99-200 Poddebice, Poland; bilinski_wojtek@wp.pl; 3 Department of Surgical and Perioperative Sciences, Division of Orthopaedics, Sunderby Research Unit, Umeå University, 971 80 Luleå, Sweden; przemyslaw.paradowski@umu.se; 4 Clinical Epidemiology Unit, Orthopedics, Department of Clinical Sciences Lund, Lund University, 223 62 Lund, Sweden

**Keywords:** fatty liver disease, metabolic risk, enzymes

## Abstract

Introduction: Metabolic dysfunction-associated fatty liver disease (MAFLD)—a new definition for non-alcoholic fatty liver disease—reflects the impact of metabolic abnormalities on liver function. We assessed the diagnostic accuracy of biomarker-based scores for prediction of MAFLD in apparently healthy children. Methods: This study included 144 children aged 9–11. MAFLD was recognized in 14 girls and 29 boys. Anthropometric indices, glycemia, insulin resistance, lipid profile, enzymes (ALT, AST, GGT, ALP), CRP, *N*-terminal propeptide of type I procollagen (P1NP) and collagen type I C-telopeptide (CTX-1) levels were measured. Fatty liver and hepatic steatosis index (FLI, HSI) and potential indicators of liver fibrogenesis: P1NP/ALP, P1NP/ALPxALT, P1NP/ALPxCRP were calculated. Results: P1NP/ALPxALT and P1NP/ALPxCRP were significantly higher in subjects with MAFLD. FLI was a good, significant predictor of MAFLD occurrence, regardless of sex. In boys, P1NP/ALPxCRP was a comparable predictor as CRP (OR 1.14 vs. 1.17; *p* < 0.001). P1NP/ALPxCRP had better discrimination capability in boys (AUC = 0.79; *p* < 0.001). However, the use of this algorithm did not improve discriminatory power in comparison to CRP (AUC = 0.81; *p* < 0.001), but gave a better sensitivity for MAFLD prediction (86% vs. 59%). Conclusions: We suggest that P1NP/ALPXCRP is a reliable tool for MAFLD prediction in routine pediatric practice.

## 1. Introduction

Obesity-associated non-alcoholic fatty liver disease (NAFLD) is considered the most common liver disease in children with an estimated prevalence of 22.5% [1,2]. A new definition—metabolic dysfunction-associated fatty liver disease (MAFLD)—has been suggested by the International Expert Consensus Panel as being more representative of metabolic disturbances related with NAFLD [1,3]. MAFLD occurs in European children and adolescents with excess body weight at 24.2% with a predominance among boys [2,4]. However, MAFLD may also occur in non-obese children with an approximate frequency of 8% [2,3,4]. 

Diagnosis of MAFLD in children combines the criteria enabling the detection of liver steatosis based on histological, imaging techniques or activity of liver enzymes, with the excess adiposity, presence of prediabetes and two or more features of metabolic syndrome [5]. The new definition allows for better identification of subjects with metabolic abnormalities being at higher risk of progression from liver steatosis to fibrosis [6]. Though liver biopsy, an invasive procedure fraught with risk of complications, remains the diagnostic gold standard for staging of NAFLD or non-alcoholic steatohepatitis (NASH), it should be avoided in children [7]. 

Non-invasive assessment of liver fibrosis relies on imaging techniques and/or different algorithms including biochemical markers of fibrosis and/or linked to fibrogenesis [8]. The features of NAFLD in children are different in comparison to adults with more likely steatosis and steatohepatitis; however, the elastography-based techniques have proven to be suitable also in the pediatric population for determination the stage of fibrosis [7,9,10]. Measurement of liver stiffness with transient elastography is suggested as a promising procedure for screening and monitoring fibrosis in pediatric NAFLD [6]. 

Several non-invasive methods based on the assessment of biochemical markers were proposed for the diagnosis of NAFLD with a particular focus on the detection of early fibrosis [9,10]. The combination of blood biomarkers with clinical features may improve the sensitivity of the scoring systems; however, their predictive value depends on the characteristics of studied population [11].

Recently, serum biomarkers of inflammation (IL-6) and extracellular matrix components (*N*-terminal propeptide of type I procollagen (P1NP), Mac-2 binding protein glycosylation isomer (M2BPGi), type III procollagen (PIIINP) and hyaluronic acid) generated during fibrogenesis, were indicated as useful for determination the stages of NAFLD in children and adolescents [9,12]. Both PIIINP and hyaluronic acid were assessed as new promising biomarkers in obese children with NAFLD [12]. P1NP/osteocalcin ratio or P1NP/alkaline phosphatase ratio along with alanine aminotransferase showed good diagnostic ability in predicting early fibrosis in children and adolescents with ultrasonography-proven NAFLD [9]. 

The aim of our study was to assess the diagnostic accuracy of existing and new serum biomarker-based scores, with special regard to biomarkers of steatohepatitis (inflammation and fibrogenesis), for prediction of MAFLD risk in apparently healthy children.

## 2. Materials and Methods

This cross-sectional study included 144 children aged 9–11, randomly selected from a cohort of 284 primary school children enrolled in a previous study [13]. In the study group, MAFLD was recognized in 43 subjects, based on the below described criteria. Children did not receive any supplements or medicines which might have affected their bone metabolism.

The study was conducted in accordance with the Declaration of Helsinki, and approved by the Ethics Committee of Nicolaus Copernicus University in Torun, Collegium Medicum in Bydgoszcz, Poland (no. KB 338/2015, annex 487/2019). Parental written consent forms were obtained for all participants before inclusion in the study.

### 2.1. Laboratory and Anthropometric Measurements

Fasting venous blood was collected after at least 8 h of fasting. Plasma glucose and serum triglycerides (TG), HDL-cholesterol (HDL-C), C-reactive protein (CRP), insulin, alanine and aspartate transaminase (ALT, AST), gamma-glutamyl transferase (GGT) and alkaline phosphatase (ALP) were measured on an Alinity ci-system (Abbott Laboratories Inc., Abbott Park, IL, USA). Enzyme activity was assayed in duplicates from each individual in the same run. Each sample underwent, in advance, an assessment of hemolysis index to exclude an interference in ALT, AST and ALP activity.

*N*-terminal propeptide of type I procollagen (intact-P1NP), collagen type I C-telopeptide (CTX-1) were assayed in deep frozen serum on an IDS-iSYS automated platform with the use of chemiluminescence technology (Immunodiagnostic Systems Holding PLC, Boldon, UK) [14]. Insulin resistance (HOMA-IR) and bone turnover index (BTI) were assessed as previously described [14]. Age- and sex-adjusted i-P1NP and CTX-1 Z -scores were calculated to derive a bone turnover index. Positive BTI, a value derived by subtracting the CTX z-score from P1NP z-score, indicated the predominance of bone formation over bone resorption. The fatty liver index (FLI) and hepatic steatosis index (HSI) were also calculated as described in the literature [15]. All laboratory tests were performed at the Department of Laboratory Medicine, Collegium Medicum, Nicolaus Copernicus University in Torun, Poland.

Anthropometric measurements were performed on the same day as blood was taken and blood pressure was measured. BMI (body mass index) and BMI percentiles were calculated with the use of an online calculator for children and adolescents in Poland as reported earlier [16].

### 2.2. Decision Criteria for Study Participants

The features of MAFLD were attributed in accordance with the International Expert Consensus Statement [1,3]. Participants were divided into two groups: with suspected MAFLD (MAFLD+), presenting with liver enzymes activity (GGT, ALT, AST) over the upper reference limit (URL) for age and sex, overweight/obesity and/or central obesity plus any other two or more features of metabolic dysregulation (low HDL-C, elevated TG and/or TG/HDL-C, elevated SBP) and a group without MAFLD. Pediatric reference intervals for age and sex, related to the instrumentation and applied methodology, with URL ≥ 22 IU/L and ≥24 for ALT in girls and boys, GGT ≥ 16 IU/L and AST ≥ 36 IU/L were accepted according to Adeli et al. [17]. BMI percentile classifications were performed in accordance with the guideline: overweight ≥ 85 and <95; obesity ≥ 95 percentile [13]. Central obesity was defined by a waist circumference (≥90th percentile of WC by sex and age for European population), increased blood pressure as SBP ≥ 90th percentile of BP by sex, height and age [13]. Fasting dyslipidemia was defined as TG ≥ 110 mg/dL and HDL-C < 40 mg/dL, fasting hyperglycemia as glucose 100–125 mg/dL [13]. TG/HDL-C ratio > 2.25 was accepted as elevated [1]. Insulin resistance was recognized as HOMA-IR at a cut-off value of ≥3.0 [18].

### 2.3. Statistical Analysis

The data were presented as medians and 25th and 75th percentiles (non-Gaussian distribution). The Shapiro–Wilk test was applied to test the normality. The variables were compared using the Mann–Whitney U test. To test for the significance of difference between two percentages or correlation coefficients we used the chi-square (Fisher exact) test. Spearman rank correlations were evaluated. Parameters with non-Gaussian distribution were normalized by natural log transformation for and univariable logistic analysis. In all the logistic models, odds ratios (ORs) were calculated for a 1 unit increase in independent variables. The significance of coefficients in the logistic models was tested using Wald chi-squared statistics. The goodness of fit of the models was evaluated using the Hosmer and Lemeshow chi-square test. Additionally, ROC curves were constructed for single laboratory parameters and the area under curve (AUC) was calculated with a 95% confidence interval (95% CI). Sensitivity, specificity negative and positive predictive values (NPV and PPV) were also calculated. The level of statistical significance was set as 0.05. Statistical analysis was performed using Statistica 13.3 software (StatSoft Inc., Tulsa, OK, USA). The level of significance was set as 0.05.

## 3. Results

### 3.1. General Characteristics of Study Group

Clinical and biochemical characteristics of study groups, stratified by sex, are displayed in Table 1. In our overall study group, 29.9% children presented characteristics of MAFLD and the number of boys was twice as high as girls. In general, girls and boys had similar SBP and ALP regardless of MAFLD occurrence. Among the most frequent features of MAFLD elevated GGT, AST and excessive body mass with central obesity were characteristic of girls whereas elevated ALT and TG/HDL-C occurred less frequently. In boys, the features most characteristic of MAFLD were excessive body mass with central obesity, elevated GGT and TG/HDL-C. Elevated AST occurred less frequently and elevated ALT was found rarely in boys. CRP levels ≥ 2.0 mg/L were found much more frequently in boys MAFLD (+) than in girls. Moreover, FLI and HSI values were significantly higher (*p* < 0.001) in children with suspected MAFLD.

### 3.2. Biomarker-Based Scores for Prediction of MAFLD Occurrence

In the whole study group, the concentration of P1NP (a marker of bone formation) was higher in girls than in boys (*p* = 0.005) but levels of CTX (a marker of bone resorption) were similar. BTI was also higher in girls (*p* = 0.011).

Positive values of BTI found in children with MAFLD indicated the predominance of P1NP over CTX release into the circulation. In all MAFLD (+) children, significant positive Spearman rank correlation was found between BTI and GGT (r = 0.508; *p* = 0.01). Strong correlations between P1NP and ALT or HDL-C (r = 0.686, *p* = 0.007; r = −0.74, *p* = 0.003) were observed exclusively in girls with MAFLD. However, P1NP/ALP ratio did not correlate significantly with metabolic features in girls who were MAFLD (+). 

In this study, we applied several new prediction scores to assess the process of fibrogenesis and inflammatory characteristics of steatohepatitis such as P1NP/corrected by ALP, P1NP/ALPxALT, and P1NP/ALPxCRP. It needs to be underlined that suspected MAFLD did not significantly affect the levels of P1NP, CTX and the P1NP/ALP scores which were similar regardless of sex (Table 2). However, when the groups MAFLD+ (*n* = 43) and MAFLD− (*n* = 101) were compared, the P1NP/ALP score was significantly lower (7.3 (5.7–8.9) vs. 8.3 (6.4–11.0) *p* = 0.034) in MAFLD+ children. It is worth pointing out that values of all potential indicators of steatohepatitis (P1NP/ALPxALT and P1NP/ALPxCRP) were found to be significantly higher in children with MAFLD (Table 2).

The analysis of Spearman correlations performed in boys and girls showed significant relationships between P1NP/ALPxALT and WC, better in boys than in girls (r = 0.389; *p* < 0.001 vs. r = 0.233; *p* = 0.045). Moreover, in boys and girls, good and highly significant correlations of P1NP/ALPxALT with FLI were found (r = 0.508; *p* < 0.001 and r = 0.358; *p* = 0.002). P1NP/ALPxCRP correlated significantly with BMI centile, FLI, and HSI and weakly but significantly with ALT, AST, TG and TG/HDL-C (r = 0.51, r = 0.47, r = 0.49, respectively, all *p* < 0.001 and r = 0.33, r = 0.28, r = 0.24, r = 0.30, all *p* < 0.05) in boys. In girls, significant correlations were found for BMI centile, FLI, HSI and ALT (r = 0.46, r = 0.39, r = 0.42, r = 0.38, respectively, all *p* < 0.001) and weaker correlation for TG/HDL-C and glucose (r = 0.28 and r = −0.0.25, all *p* < 0.05). 

Next, we performed logistic regression analysis for evaluation of the relationship between biomarkers, indicators of steatohepatitis and the presence of MAFLD (Table 3). Only those variables significantly different in the comparative analysis and not included in the MAFLD classification criteria were applied (except FLI and HSI as well-documented algorithms). Sex-specific differences were found in terms of the predictors of MAFLD. The univariable analysis indicated that P1NP/ALP was not a predictor of MAFLD regardless of gender. 

Other evaluated biomarkers showed gender-specific differences in the prediction power. In boys, FLI, CRP, P1NP/ALPxCRP, HOMA-IR and HSI were significant positive predictors of MAFLD. By contrast, in girls, only FLI and HSI showed significant positive prediction power for MAFLD presence. 

### 3.3. Diagnostic Performance of Biomarker-Based Scores for Prediction of Metabolic (Dysfunction)-Associated Fatty Liver Disease (MAFLD)

We performed ROC analysis in the study group with regard to sex, to assess the diagnostic capability of evaluated scores in terms of sensitivity, specificity, positive and negative predictive values (Table 4). The P1NP/ALP ratio had no predictive value for MAFLD in our study group. FLI and HSI alone had a very good diagnostic accuracy in our cohort to distinguish between subjects with and without MAFLD. It is noteworthy that FLI performed much better when evaluated in boys. Both CRP and HOMA-IR had a better discrimination power in boys. The use of prediction parameters in combination, such as P1NP/ALPxCRP, did not improve discrimination capability but yielded a better sensitivity in boys.

## 4. Discussion

The increase in prevalence of metabolic abnormalities associated with fatty liver disease among overweight and obese children has implications for long-term health. Accurate diagnosis of metabolic dysfunction-associated fatty liver disease has received growing attention since an International Consensus provided the criteria of pediatric MAFLD [1].

A recent cross-sectional study on the prevalence of MAFLD in Finnish children and adolescents aged 2–16, examined due to overweight, reported that MAFLD was present in 18% of boys and 11% of girls [19]. However, in this study, MAFLD was recognized based only on the elevated levels of ALT. In our study on apparently healthy children aged 9–11 years, MAFLD was recognized in 29.9% of subjects and more often in boys than in girls. The frequency of MAFLD was similar to that reported by others in pediatric populations [1,4]. 

A very recent large sample study showed that the prevalence of NAFLD among 10-years-old obese children reached 25% [20]. It has been shown that a high proportion of children with obesity may have liver steatosis or steatohepatitis with some degree of fibrosis [1,21]. In our overall study group, obesity was found in 19.4% of children but in those with MAFLD excessive body weight and/or elevated WC were identified in 79%.

The most frequent features of MAFLD in our cohort of children were elevated GGT, excessive body mass and central obesity. Elevated TG/HDL-C (≥2.25), HOMA-IR (>3.0) and CRP levels (≥2 mg/L) were characteristic of boys MAFLD (+). Elevated HOMA-IR and CRP were, so far, not included in the proposed pediatric MAFLD criteria [2]. It needs to be highlighted that the level of ALT in children with MAFLD was elevated only to a small extent. Ma et al. reported that in the pediatric population, the sensitivity of ALT as a screening test for NAFLD is low [22]; therefore, our findings are consistent with data indicating that fatty liver disease, including fibrosis, may be recognized with normal ALT in pediatric and adult populations [1,23,24].

Imaging techniques may be applied for the assessment of liver fibrosis degree and monitoring treatment [7,25]; however, recently the use of non-invasive tests was proposed as an alternative to identify subjects with early and advanced fibrosis [8]. In children, multiple prediction models based on blood biomarkers have been evaluated to distinguish stages of fibrosis as a complication of NAFLD [9,26,27,28,29]. It is worth noting that most of these prediction models were based on parameters included in the MAFLD definition applied in this study (e.g., liver enzymes, BMI, lipid parameters).

FLI and HSI algorithms are the best researched and the most popular non-invasive tests for assessment of liver diseases. FLI has been developed as a predictor of hepatic steatosis and HSI as a screening tool for NAFLD in the general adult population [30,31]. The data on FLI and HSI performance for assessment of liver steatosis in children are scarce. Both FLI and HSI were evaluated in overweight and obese children, at the similar age as in our study, and proved useful as non-invasive biomarkers of liver steatosis [25,26]. 

In the present study, we assessed the usefulness of non-invasive serum biomarker-based algorithms for prediction of MAFLD in apparently healthy children. We compared the diagnostic accuracy of existing and well-known algorithms (FLI, HSI) and new algorithms (P1NP/ALP, P1NP/ALPxCRP) for prediction of MAFLD with presumptive fibrogenesis and inflammation. 

We did not assess the diagnostic accuracy of other algorithms (P1NP/ALPxALT) proposed by others as ALT was used in our study for group stratification [9]; in addition, P1NP, a collagen type 1 turnover product, was indicated as useful for the prediction and monitoring of liver fibrosis in adults [32]. Its increased levels were found to be associated with advanced stages of fibrosis in obese subjects with diabetes and MetS [32]. A recent study on adolescents with NAFLD, diagnosed by ultrasonography and fibroscan, suggested that serum P1NP and IL-6 along with routine chemistry tests can be used as predictors for assessing steatohepatitis and fibrosis [9].

When assessing different formulas, we also applied those including serum level of P1NP released during collagen remodeling. In the course of fibrogenesis, collagen type 1 metabolism in the liver markedly increases leading to release of P1NP which can be measured as a marker of hepatic collagen formation [9,33]. However, it should be considered that collagen type 1 turnover is characteristic for bone and liver and, notably in childhood, bone modeling is high to allow rapid bone mass accretion during growth. The level of bone turnover markers in children is high and that of P1NP peaks at 13–14 years in boys and at 11 years in girls and then gradually declines [34]. Therefore, to distinguish between the relative contribution of each tissue to the serum level of P1NP the correction for another bone formation marker is necessary. 

Our results showed that the well-known algorithms FLI and HSI are good positive predictors of MAFLD presence in both sexes, particularly in boys (AUC ≥ 0.89). Both FLI and HSI had a very high NPV values with a lower PPV values. A very good prediction power of FLI can be explained by the fact that it includes the values of TG and GGT, which in our MAFLD (+) children were mostly elevated. Others also observed a good positive prediction of HSI and FLI for hepatic steatosis in children but with slightly lower AUC values [24,35]. 

The P1NP/ALP ratio as a marker of liver fibrosis had no predictive value for MAFLD in our study group, which could potentially indicate the absence of significant hepatic fibrosis. However, we showed that children, especially boys, with MAFLD present higher CRP level and insulin resistance (HOMA-IR > 3.0). We can only hypothesize that these changes may reflect an early stage of fatty liver disease.

We also demonstrated sex-specific differences in relation to the predictors of MAFLD. In boys, CRP and HOMA-IR were better positive predictors of MAFLD presence than in girls. In the same way as CRP, the P1NP/ALPxCRP algorithm had better discrimination capability in boys. However, the use of this new algorithm did not improve discriminatory power, but gave a better sensitivity for MAFLD prediction in boys (P1NP/ALPxCRP 86% vs. CRP 59%). CRP alone was recognized as a predictor of liver steatosis and fibrosis in MAFLD subjects [36], but it is a non-specific marker of liver inflammation. Thus, we believe that the P1NP/ALPxCRP algorithm could be used as a more liver-specific test. However, this hypothesis requires further investigation in children. Interestingly, the prediction power of P1NP/ALPxCRP formula applied by us was similar to that of IL-6xAST used by others for assessing liver inflammation [9].

A limitation to this study is that our findings are based exclusively on laboratory and anthropometric data and the number of participants with MAFLD was small. The presented results necessarily require confirmation of the presence of fatty liver with non-invasive methods of recognized diagnostic value, e.g., transient elastography. 

We have nonetheless made an assumption that considering all proposed criteria for diagnosis of pediatric MAFLD allows for proper recognition [1]. The early stage of fatty liver in our study group may also be indicated by the fact that only 4.9% of subjects had FLI values ≥ 30 which is recommended as the cut-off for NAFLD in children [37]. Screening for NAFLD in obese and overweight children between 9 and 11 years of age is recommended by NASPGHAN with assessment of ALT as a screening tool [35]. We assessed ALT, AST and GGT in a homogenous group of prepubertal girls and boys, this being the strength of our study. Given the high prevalence of MAFLD in obese children and its presence in non-obese ones, the ability for diagnosis of MAFLD with the use of simple screening tests is of particular importance. Another limitation is that the study included only children in the narrow age range between 9 and 11 years. However, we decided to include prepubertal children in the study to reduce the potential impact of hormonal changes on laboratory indices related to bone metabolism. What is of importance is that the level of P1NP is quite stable between ages 9 and 11 [34].

We are aware that for the diagnostic assessment of new indicators of liver fibrosis, broader studies are indispensable, taking into consideration different stages of children’s development.

The risk of progression of liver steatosis to steatohepatitis with some degree of fibrosis in children is high [1]. To date, the noninvasive measures to assess stages of fatty liver disease in children are not well validated despite the new potential biomarker of fibrosis (PRO-C3, a propeptide released during collagen type III formation) appearing on the horizon [38].

## 5. Conclusions

We suggest that P1NP/ALPXCRP, a formula of good diagnostic accuracy, is a reliable tool for MAFLD prediction in apparently healthy children and may be useful in pediatric clinical practice.

## Figures and Tables

**Table 1:** Characteristics of study groups stratified by sex.

Variables	Girls			Boys		
	MAFLD+ (*n* = 14)	MAFLD− (*n* = 60)	*p*	MAFLD+ (*n* = 29)	MAFLD− (*n* = 41)	*p*
Age (years)	9 (9–10)	10 (9–10.5)	0.035	10 (9–10)	10 (9–11)	0.748
BMI centile	85 (64–95)	54 (19–70)	<0.001	95 (71–96)	38 (21–43)	<0.001
BMI centile ≥ 85 (%)	57.1	6.7	0.015	65.5	7.3	0.002
WC (cm)	74 (63–80)	66 (59.5–70)	0.008	78 (69–86)	63 (59–67)	<0.001
Central obesity * (%)	42.9	5.0	<0.001	41.4	4.9	<0.001
Glucose (mg/dL)	91 (86–95)	93 (87–100)	0.252	92 (85–97)	97 (91–103)	0.018
HOMA-IR	2.3 (1.8–3.5)	1.8 (1.2–2.7)	0.187	2.8 (2.1–4.4)	1.7 (1.1–2.7)	0.001
HOMA-IR > 3.0 (%)	28.6	21.7	0.581	44.8	21.9	0.042
SBP (mmHg)	110 (106–114)	110 (103–114)	0.644	116 (103–120)	109 (103–115)	0.201
TG (mg/dL)	98 (84–110)	67 (51–84)	0.002	105 (65–157)	56 (41–74)	<0.001
HDL-C (mg/dL)	53 (47–62)	60 (54–69)	0.095	51 (47–58)	61 (55–73)	0.001
TG/HDL-C > 2.25 (%)	28.5	8.3	0.037	51.7	0.0	<0.001
CRP (mg/L)	1.58 (0.46–2.63)	0.41 (0.16–1,11)	0.032	1.33 (0.49–2.60)	0.20 (0.12–0.65)	<0.001
CRP > 2.0 mg/L (%)	28.6	11.7	0.110	41.4	0.0	<0.001
ALP (U/L)	206 (161–243)	184 (145–234)	0.366	211 (155–225)	175 (148–222)	0.567
GGT (U/L)	20 (15–23)	12 (10–14)	<0.001	17 (13–21)	10 (9–13)	<0.001
GGT ** (%)	71.4	6.7	<0.001	62.1	9.8	<0.001
ALT (U/L)	16 (9–22)	6 (4–8)	<0.001	13 (8–16)	6 (3–7)	<0.001
ALT ** (%)	28.6	2.1	0.005	6.9	0.0	0.088
AST (U/L)	36 (32–40)	28 (26–30)	<0.001	32 (30–35)	29 (28–32)	0.007
AST ** (%)	50.0	3.3	<0.001	24.1	12.2	0.740
FLI	13.3 (3.1–20)	1.4 (0.9–2.5)	<0.001	11.9 (5.5–23)	0.95 (0.63–1.4)	<0.001
HSI	0.08 (0.05–0.17)	0.02 (0.01–0.03)	<0.001	0.13 (0.03–0.30)	0.01 (0.008–0.02)	<0.001

Results are presented as median (Q1–Q3) or percentage; * WC ≥ 90th percentile; ** GGT, ALT, AST ≥ URL.

**Table 2:** Biomarkers associated with steatohepatitis in the study groups.

Variables	Girls			Boys		
	MAFLD+ (*n* = 14)	MAFLD− (*n* = 60)	*p*	MAFLD+ (*n* = 29)	MAFLD− (*n* = 41)	*p*
CTX (ng/mL)	1.52 (0.96–2.15)	1.43 (1.11–2.09)	0.907	1.44 (1.05–1.92)	1.45 (1.03–1.96)	0.962
P1NP (ng/mL)	1368 (1218–1488)	1423 (1246–2481)	0.482	1289 (1208–1396)	1254 (1169–1443)	0.849
BTI	0.29 (−0.47–0.92)	0.38 (−0.25–1.20)	0.512	0.10 (−0.76–0.44)	−0.11 (−0.53–0.55)	0.990
P1NP/ALP	7.3 (5.8–10.7)	8.7 (6.4–12.4)	0.207	7.3 (5.6–8.6)	7.9 (6.0–8.8)	0.273
P1NP/ALPxALT	123 (66–169)	51 (29–90)	0.004	87 (71–120)	45 (17–80)	<0.0501
P1NP/ALPxCRP	11.81 (3.52–19.18)	3.18 (1.54–10.77)	<0.043	9.69 (3.04–17.83)	1.58 (0.81–4.71)	<0.001

Results are presented as median (Q1–Q3).

**Table 3:** Univariable logistic regression analysis for prediction of MAFLD.

Variables	Girls (*n* = 74)		Boys (*n* = 70)	
	*p*	OR (95% CI) *	*p*	OR (95% CI) *
CRP	0.042	1.02 (1.00–1.05)	<0.001	1.17 (1.08–1.27)
HOMA-IR	0.444	1.01 (0.98–1.04)	0.002	1.07 (1.02–1.11)
P1NP/ALP	0.287	0.92 (0.78–1.08)	0.315	0.92 (0.77–1.09)
FLI	<0.001	1.30 (1.14–1.49)	<0.001	1.70 (1.28–2.26)
HSI	<0.001	1.23 (1.09–1.38)	0.005	1.08 (1.02–1.13)
P1NP/ALPxCRP	0.063	1.02 (1.0–1.04)	<0.001	1.14 (1.05–1.24)

* per 0.1 unit increase in CRP and HOMA-IR; per 0.01 unit increase in HSI; per 1.0 unit increase in P1NP/ALP, P1NP/ALPxCRP.

**Table 4:** Diagnostic capability of evaluated scores for prediction of MAFLD.

Variables	Girls *n* = 74				Boys *n* = 70			
	AUC (95% CI)	Sensitivity/Specificity (%)	PPV/NPV (%)	*p*	AUC (95% CI)	Sensitivity/Specificity (%)	PPV/NPV (%)	*p*
FLI	0.85	86/75	44/96	<0.001	0.95	93/83	79/94	<0.001
	(0.74–0.92)				(0.87–0.99)			
HSI	0.84	86/87	60/96	<0.001	0.89	83/83	77/87	<0.001
	(0.74–0.92)				(0.80–0.95)			
CRP	0.69	64/75	38/90	0.038	0.81	59/90	81/76	<0.001
	(0.57–0.79)				(0.70–0.90)			
HOMA-IR	0.61	86/42	26/93	0.124	0.74	86/56	58/85	0.001
	(0.49–0.73)				(0.62–0.83)			
P1NP/ALP	0.61	64/63	29/88	0.192	0.58	62/59	51/69	0.269
	(0.45–0.77)				(0.44–0.72)			
P1NP/ALP xCRP	0.68	64/75	38/90	0.041	0.79	86/59	60/86	<0.001
	(0.56–0.78)				(0.67–0.88)			

## Data Availability

Not applicable.
